# A Predictive Model Assessing Genetic Susceptibility Risk at Workplace

**DOI:** 10.3390/ijerph16112012

**Published:** 2019-06-05

**Authors:** Pieranna Chiarella, Pasquale Capone, Damiano Carbonari, Renata Sisto

**Affiliations:** Department of Occupational and Environmental Medicine, Epidemiology and Hygiene, INAIL Research, Via Fontana Candida 1, 00078 Monteporzio Catone, Rome, Italy; p.capone@inail.it (P.C.); d.carbonari@inail.it (D.C.)

**Keywords:** biomonitoring, ethnicity, gene polymorphism, occupational health, migration, susceptibility biomarker

## Abstract

(1) Background: The study of susceptibility biomarkers in the immigrant workforce integrated into the social tissue of European host countries is always a challenge, due to high individual heterogeneity and the admixing of different ethnicities in the same workplace. These workers having distinct cultural backgrounds, beliefs, diets, and habits, as well as a poor knowledge of the foreign language, may feel reluctant to donate their biological specimens for the biomonitoring research studies. (2) Methods: A model predicting ethnicity-specific susceptibility based on principal component analysis has been conceived, using the genotype frequency of the investigated populations available in publicly accessible databases. (3) Results: Correlations among ethnicities and between ethnic and polymorphic genes have been found, and low/high-risk profiles have been identified as valuable susceptibility biomarkers. (4) Conclusions: In the absence of workers’ consent or access to blood genotyping, ethnicity represents a good indicator of the subject’s genotype. This model, associating ethnicity-specific genotype frequency with the susceptibility biomarkers involved in the metabolism of toxicants, may replace genotyping, ensuring the necessary safety and health conditions of workers assigned to hazardous jobs.

## 1. Introduction

Italy is a small country with a long history of emigration and a very short experience with immigration. In the past fifty years, Italy has experienced massive waves of migrations of different ethnicities, shifting from an emigration to an immigration country in the year 1970. The first immigrants to settle down were Europeans, while from 1990 to 2000 there was a substantial increase in foreign citizens coming from Asia and Africa. In those years, a concomitant rise in eastern European immigrants was also registered, and almost 700,000 non-European Union (EU) workers were legalized [1]. 

The integration of immigrants into our social tissue showed that our country is mostly characterized by young and less-educated immigrants employed in traditional working sectors, such as cleaning services, manufacturing, naval industries, and agriculture [1,2]. Chinese and Filipinos have been employed, respectively, in commercial services (restaurants, shops) and cleaning activities, or in companies and private houses, while citizens of eastern Europe (i.e., Albania, Romania, Estonia, Ukraine) have managed to find jobs in the construction, house renovation, and caregiver sectors [3]. In addition, Bangladeshis and Nigerians, predominant in the north and center of the country, have been recruited mainly in manufacturing activities, naval industries, and agriculture. 

**Figure 1:**
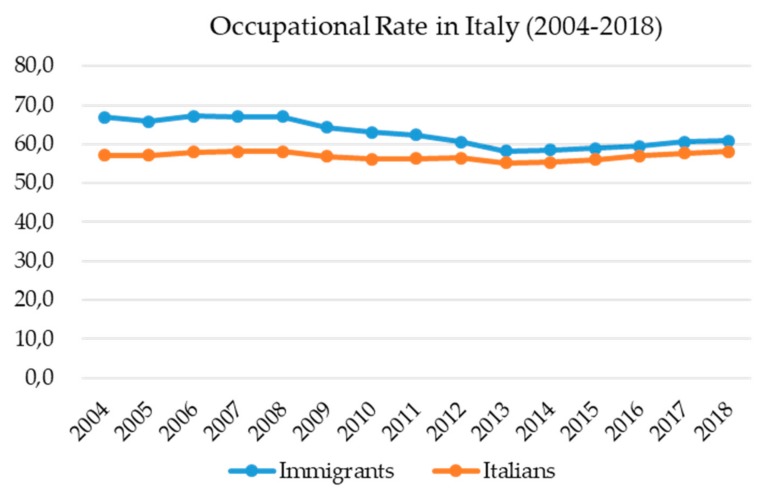
This graph shows the occupational rate in the years 2004–2018 of immigrant and Italian workers with resident permits.

To date, the immigrant workforce having a regular resident card in Italy indicates an employment rate of 59% against a 9.7% unemployment rate, which parallels and even overcomes the local Italian workforce (Figure 1) [3]. Therefore, despite the fact that Italy is not a wealthy country, which is demonstrated by the difficulties for Italians in finding a job, several opportunities have been offered also to immigrants. In particular, specific ethnicities, such as the small populations coming from Nigeria, Bangladesh, and Senegal, have been recruited in occupational sectors where Italians nowadays represent a small minority. This is why it is important to highlight the similar chance in job opportunities between local and migrant workers (Figure 1). 

Several jobs, particularly those related to the manipulation of toxic and carcinogenic agents, include volatile organic solvents, such as styrene (International Agency for Research on Cancer (IARC) group 2A), toluene and xylene (IARC group 3), and ethylbenzene (IARC group 2B), used in the naval industries; formaldehyde (IARC group 1), used in anatomy and pathology laboratories; benzene (IARC group 1), commonly found in the petrochemicals industry; and metals employed in several manufacturing companies. All of these toxicants expose workers to chemical risk, which is dangerous for their health and safety [4,5,6,7,8]. The toxic nature of these substances is dependent on their type of toxicity, as well as on their potential damage on the human organism, whereas the intensity and duration of the toxic responses are dependent on the final concentration of the toxicant accrued in the target organ. This is closely related to a lower or a higher efficiency of the individual metabolism, which consists of absorption, distribution, biotransformation, and elimination of the toxic agent, and depends on his or her genetic ancestry [9]. 

Many studies showed that single nucleotide polymorphisms (SNPs) may affect the detoxification pathway of dangerous chemical agents, reducing or increasing their toxicity [10]. In particular, defective gene polymorphisms of enzymes involved in the metabolism of these compounds may cause the absence or reduction of activity, determining a variable fluctuation in the urinary metabolite excretion with potential negative effects on the worker’s health [11].

However, the exposure risk to these substances is strictly controlled by the employer company, which must verify worker observance of occupational exposure limits (OELs), the wearing of personal protection equipment (PPE), and the scheduling of periodical health surveillance and monitoring. 

Apart from mandatory surveillance, some companies might wish to join in facultative environmental and biomonitoring campaigns, proposed as investigational studies promoted by Italian Workers’ Compensation Authority (INAIL), whose tasks are workers compensation, health prevention, and protection at workplaces. The research proposal has to be approved by the company and by the regional authorities. The aim is to follow up, in a defined period of time, the indoor exposure risk of workers and of relative controls during the programmed work shifts. 

Traditionally, potential health risks are assessed by environmental and biological monitoring. The first measures are the indoor levels of toxic agents (i.e. environmental biomarkers); the second detect (1) the subject-absorbed dose of toxicant (biomarkers of dose or exposure), (2) the genotoxic damage (biomarkers of effect), and (3) the individual gene polymorphisms associated to the metabolism of dose biomarkers (biomarkers of susceptibility) [6]. Hence, once the biomarkers of dose, early effects, and susceptibility have been measured, a snapshot of the exposure trend of all workers is achieved in a narrow time interval. In particular, the individual response to the exposure is influenced by the genetic differences observed in the metabolism of the subjects. 

It is widely known that exposure to carcinogenic chemicals and the respective individual metabolic response are not identical for everyone, but they are commonly associated with individual metabolizing capability, which may vary among subjects within the same population and among different ethnic groups characterized by specific genetic ancestries. The identification of genetic variants that could predispose different ethnic groups to toxicants and to carcinogenic agents is considered a relevant issue in the occupational context, in order to protect the workers who may have inherited different and specific genetic susceptibilities.

As volunteers, workers exposed to hazardous substances might wish, but cannot be forced, to join the biomonitoring campaign concerning the occupational health risk assessment. A detailed questionnaire is provided to all of the participants to assess their nutritional and smoking habits, drug assumption, and presence of chronic and acute diseases, in order to consider all of the possible confounding factors affecting the experimental results.

Any test is sub-conditioned to the final objective of the study, previously approved by the local health unit, as well as by the research institute, with the informed consent of workers to donate their biological samples (urine, saliva, and blood) for downstream analysis.

The institute directing the environmental and biomonitoring campaign ensures analysis of grouped data, anonymity and confidentiality, lack of disclosure in public databases, and data exclusivity and availability to medical staff of the company, if required for internal use. All the results of the environmental and personal biomonitoring are matched and analyzed at the epidemiological level, to identify external and internal exposure levels, potential DNA damage, and the genetic susceptibility of workers. Such data may end up in a scientific publication, respecting all the criteria and principles cited above.

However, organizing a campaign is a challenging task, and there might be some critical issues that might need to be solved. To make an example, there are some migrant populations who have different behaviors, culture, diet, habits, and beliefs with respect to the local population, and therefore are unused to the standard medical procedures of the biomonitoring (i.e., programmed urine harvesting, venipuncture, genotyping analysis). In particular, the invasiveness of the venipuncture may strongly discourage workers from donating blood, which is necessary to execute individual genotyping. Other factors hindering the gene polymorphism screening include excessive financial costs sustained by the research institute to support data analysis on large numbers of individuals, the need for trained medical staff, the storage and transport of bio-samples, and the possible infection risk of personnel, as well as the necessity for official approval and authorization by the public organization to proceed with the campaign. All these details might turn the investigational study of dose, genotoxic, and susceptibility biomarkers into something almost impracticable. 

To overcome the issues discussed above, and to study the impact of the gene polymorphisms on the metabolism of carcinogenic substances, we have proposed and elaborated on a theoretical model based on statistical and principal component analysis (PCA) [12,13], which allows the use of ethnicity-specific polymorphism gene frequency to predict the susceptibility risk of defined ethnic groups. The model has been conceived as potential alternative to the genetic analysis on workers’ bio-fluids, to provide a susceptibility risk estimate in ethnicities exposed to toxicants found in the workplace.

## 2. Materials and Methods

Genotype and allele frequencies have been downloaded from the Ensembl project of genome databases for vertebrates and other eukaryotic species public database [14]. The statistics has been carried out by using R free software for statistical analysis and PCA. 

Gene polymorphisms with single nucleotide substitution (SNPs) have been selected on the basis of the exposure to toxic and carcinogenic substances commonly found in different manufacturing companies specializing in fiberglass production, electrical insulation, welding, and ship construction. To simplify the analysis, we provided a list of gene polymorphisms involved in three different aspects of human metabolism (Table 1). 

All the genes analyzed are single nucleotide polymorphisms (SNPs), where the nucleotide substitution may be comprised either in the coding sequence or in the promoter or regulatory region of the gene. The model has been designed to assess the relative risk in Africans, East Asians, South Asians, and Europeans, with respect to the worldwide population.

All the statistical analyses were performed using the statistical software SPSS (version 25, IBM, Armonk, New York, United States) and R (version 3.5.3 (2019-03-11); R Foundation for Statistical Computing, Vienna, Austria).

The relative risk (RR) = *VGF* (*eg*)/*VGF* (*all*), where *VGF* stands for variation of gene frequency, *eg* stands for ethnic group, and *all* stands for the worldwide population, has been calculated for each ethnic group as the ratio between the homozygous variant frequency of the considered ethnic group, *VGF*(*eg*), and the homozygous variant frequency of the general population, *VGF*(*all*). If *VFG*(*eg*) > 1, the risk is obviously increased in that specific ethnic group with respect to the general population. The most unfavorable condition, if not otherwise specified, is generally associated with the variant (i.e., mutant) condition, although in very few exceptions it might be associated to the wild-type genotype. 

Such exceptions have been considered in the case of four gene polymorphisms (EPHX1 rs223492, CYP2E1*6 rs6413432, CYP2E1*5B rs3813867, and *MPO rs2333227*) where the variant frequency might be advantageous relative to a single and specific step of the biochemical pathway. In such cases, the highest risk is for the wild-type frequency, and RR = *WTGF* (*eg*)/*WTGF* (*all*) is calculated as the ratio of the homozygous wild-type frequency of the ethnic group (*WTGF*(*eg*)) with respect to the homozygous wild-type frequency of the whole population (*WTGF*(*all*)). If the variant allele is advantageous, then the wild-type allele is the less favorable, followed by the heterozygous genotype showing an intermediate risk level. The RR of the heterozygous genotype has been worked out for each metabolic enzyme in the four ethnicities. Genotype, allele frequency, and RR have been reported in Supplementary Table S1.

A PCA transformation was applied for each group of genes (i.e., detoxification, oxidative stress, and DNA repair), and the ethnic groups were represented in the PC (principal component) plane of each group. The PCA is a statistical technique that uses an orthogonal transformation to convert a set of observations of a certain number of possibly non-independent variables into a set of linearly independent variables called principal components.

The transformation is performed along the directions of the eigenvectors of the covariance matrix, so that the principal components are ordered according to the eigenvalues of the covariance matrix in descending order. In other words, the first component corresponds to the maximum eigenvalue of the covariance matrix, the second component corresponds to the immediately-following eigenvalue, and so on. Usually, with the two first principal components, one is able to explain a large fraction of the variance of the data. The advantage of this technique is that the original multidimensional space of variables can be reduced to a simple plane on which the data can be easily represented. PCA is particularly useful to discriminate different groups, as they appear in separate regions of the PC plane. Both the cases and the original variables can be represented in the PC plane. For each ethnic group, the relative risk of mutation with respect to the general population was considered for each of the selected genes of the three groups: detoxification (8 genes), oxidative stress (6 genes), and DNA repair (7 genes). When the ethnic groups are close to each other in the PC plane, it means that they are similar from the point of view of the linearly independent variables, explaining most of the variance of the data. Conversely, groups that appear in different sectors of the plane are effectively discriminated by the PCA.

## 3. Results

**Figure 2:**
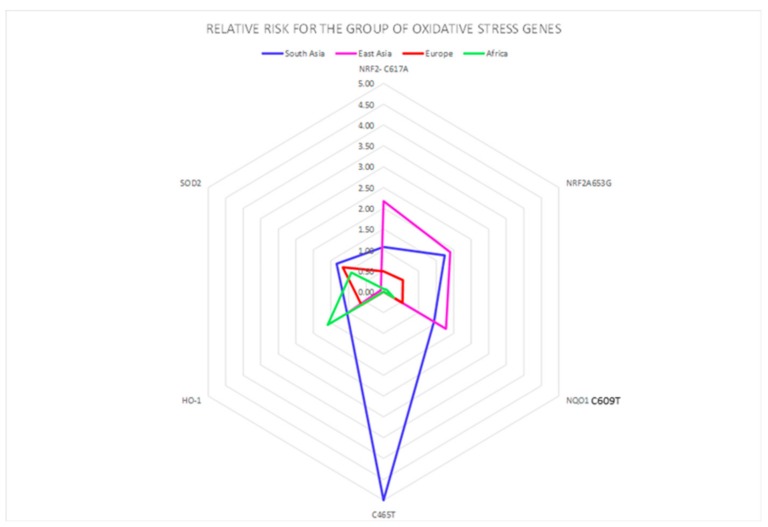
Oxidative stress polymorphic genes are highlighted with different colors in the four ethnic groups. The genes are represented by geometrical figures, where the tip of the figure points toward the most susceptible genes belonging to each gene group.

**Figure 3:**
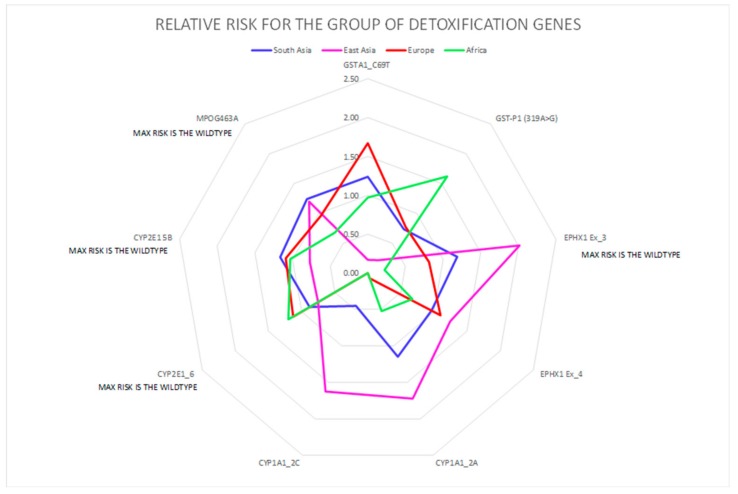
Detoxification polymorphic genes are highlighted with different colors in the four ethnic groups. The genes are represented by geometrical figures, where the tip of figure points toward the most susceptible genes belonging to each gene group.

**Figure 4:**
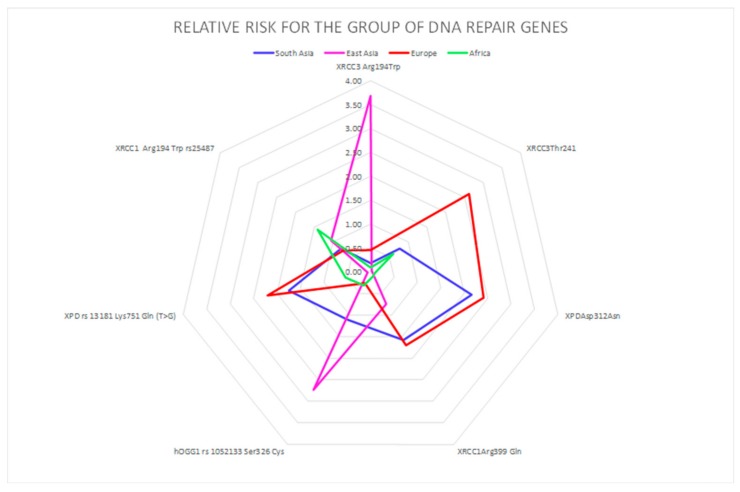
DNA repair polymorphic genes are highlighted with different colors in the four ethnic groups. The genes are represented by geometrical figures, where the tip of figure points toward the most susceptible genes belonging to each gene group.

**Figure 5:**
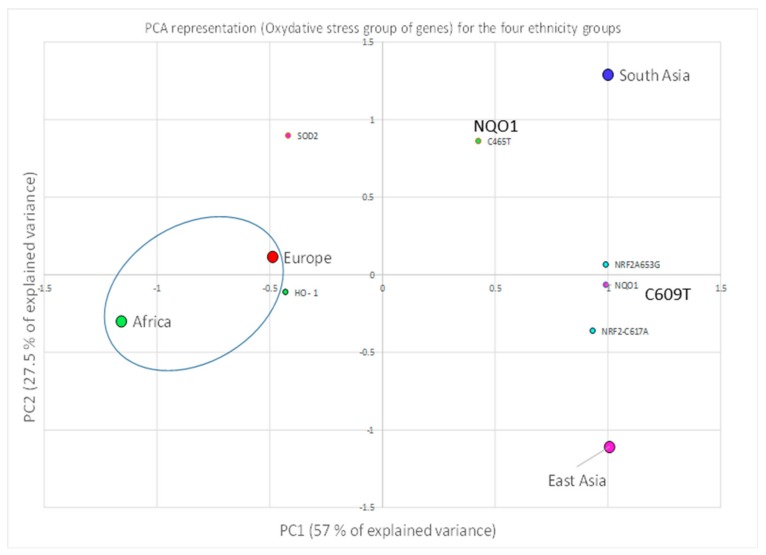
PCA representation of the relative risk of unfavorable mutation of the genes belonging to the group of oxidative stress. Both the ethnicity groups (cases) and the genes (original variables) are represented in the PC plane.

**Figure 6:**
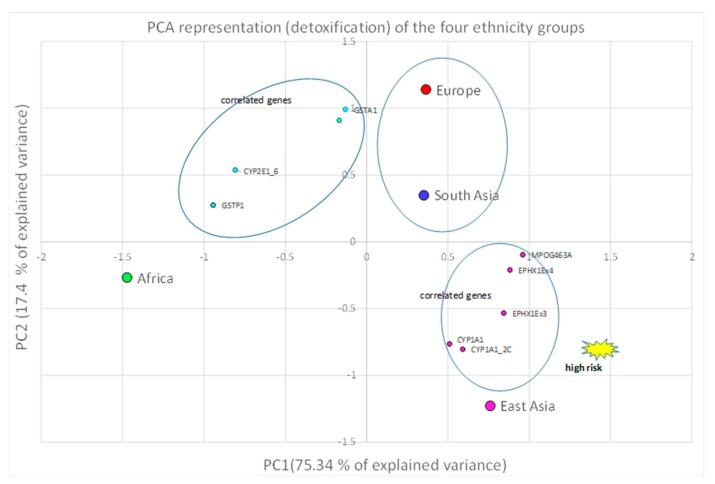
Europe and South Asia show a low-risk similarity in their detoxification genes, while East Asia is associated with a high risk for this gene group.

**Figure 7:**
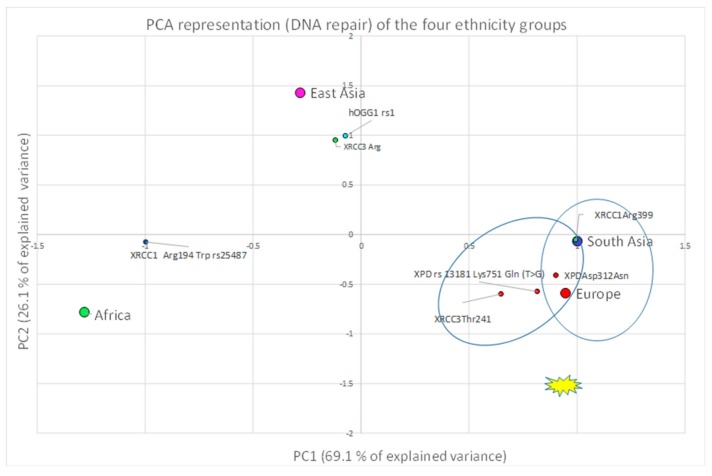
Europe and South Asia show a high correlation and high susceptibility risk with their DNA repair genes.

A correlation between the four investigated ethnicities, as well as between ethnic and polymorphic genes involved in different metabolic functions, has been worked out and visualized by PCA (Figure 2, Figure 3 and Figure 4; Figure 5, Figure 6 and Figure 7). This methodology allows us to visibly appreciate how much the four ethnic clusters are distinguished by the relative risk of the variant condition for the three metabolic gene polymorphism groups.

In Figure 2, the calculated RR for each ethnicity is visualized for the oxidative stress gene group, showing the highest susceptibility for specific genes. Similarly, the RR for each ethnicity is visualized for the detoxification gene group (Figure 3) and for the DNA repair gene group (Figure 4). The area subtended by each ethnicity curve of Figure 2 was calculated. The different areas obtained for each ethnicity were divided by the minimum area, representing the minimum risk for each of the considered groups of genes. The same procedure was repeated for Figure 3, Figure 4 and Figure 5. 

The PCA allowed us to appreciate the similarity found between ethnicities, for example in the African and European cluster (see the ellipse), which showed a high similarity and low RR for the oxidative stress genes (Figure 5). A low RR similarity between Europeans and South Asians in relation to the detoxification genes was found (Figure 6), whereas in the case of East Asians a good correlation of the ethnic group with five detoxification genes was detected, and was highlighted as high-risk (Figure 6). 

Lastly, a correlation between Europeans and South Asians in relation to four DNA repair genes was obtained. Both ethnic groups present a high risk for these genes in comparison to the other ethnicities (Figure 7).

To summarize, the comparison between the three groups of genes—detoxification, oxidative stress, and DNA repair—allowed us to identify Africans as the ethnic group at lower risk. The odds ratio with respect to Africans was then calculated, and the results are shown in Table 2. For simplicity, a cumulative index was also considered, in which the relative weight is the same for the three groups of genes. 

The PCA analysis allows us to visibly appreciate how effectively the four ethnicity groups are discriminated by the relative risk of the variant condition for the different genes of the group of genes under investigation. Africa and Europe are similar with respect to the oxidative stress genes, showing a low susceptibility risk. 

## 4. Discussion

In the context of environmental and occupational exposure, the role of biotransformation enzymes is to ensure efficient detoxification of endogenous and exogenous compounds by specific biochemical pathways. These enzymes modify dangerous substances into inactive and easily excretal forms to avoid accrual and harm to the human organism. Although the screening of individual gene polymorphisms is the ideal procedure to assess a specific susceptibility, there are several important issues mentioned in the introduction that need to be considered before starting a biomonitoring campaign, including the genotype analysis of workers.

The most critical condition that may hamper the biomonitoring campaign is represented by the lack of workers’ consent to venipuncture or urine harvesting, accompanied by the misperception of worker inability or disease, in the case of unfavorable results achieved after measuring dose, early effect, and susceptibility biomarkers. However, bearing in mind that data are analyzed in aggregated form and provided to the medical personnel for specific internal use, it is important to underline that gene polymorphism analysis has no diagnostic value, and is used only to identify the ethnic differences in polymorphisms, to discover inter-individual variation in response to exposure to various toxicants and carcinogens. Such variation is not rare, and exists because of the diversity in the human genetic background or ancestry. Furthermore, polymorphic variants are not completely fixed, but may change in the future as a consequence of population admixture and environmental selective pressure [15,16].

The efficiency in the biotransformation of specific enzymes is worthy of note. In the investigational studies carried out at the workplace, the genetic background of a worker may affect the individual’s biochemical response to the exposure. A typical example is benzene toxicity, where lowered or absent *NQO1* activity due to the presence of the NQO1*2 variant allele encodes a non-synonymous mutation (P187S) with negligible enzymatic function. This variant can increase the risk of adverse health effects, including bone marrow toxicity, after environmental exposure to benzene and benzene-like compounds [17].

Similarly, mutations in *GST-M1*, *GST-T1*, and *GST-P1* polymorphic genes can alter benzene biotransformation and the level of biomarkers of the internal dose. In *GST-T1* and *GST-M1* null genotypes, the levels of S-phenyl-mercapturic acid metabolite (SPMA) are low or absent, whereas an increase in the other biochemical pathway may lead to a higher excretion of trans,trans-Muconic acid (tt-MA) [18,19,20]. In addition, the defective function of DNA repair genes, such as *hOGG1* and *XRCC1*, may be deleterious in the typical exposure to anti-neoplastic drugs, which are handled and administered to the patients by the hospital nurses. Such working activity may expose the health care personnel, including the cleaning staff, to the risk of exposure to toxic effects, resulting in skin rash, nausea, and allergies, as well as to the risk of genotoxicity in human lymphocytes [21].

The determination of gene polymorphisms and their matching enzymes may or may not be beneficial to prevent or reduce adverse effects in response to toxicants. For instance, in our search we identified some polymorphic genes (EPHX1 rs2234922, MPO rs2333227, CYP2E1*6 rs6413432, and CYP2E1*5B rs3813867) that, at least in one step of the biochemical pathway, may not be beneficial for the homozygous wild-type genotype, which is in contrast to the assumption that the homozygous variant allele is always the most adverse condition [22,23,24,25].

However, it is noteworthy the enzyme–metabolite relationship is not linear or unidirectional, but may be multidirectional. Nonetheless, the complexity of gene–gene and gene–exposure interaction makes it difficult to identify a univocal role for each polymorphism. In the case of slow metabolizers or poor responders, compensation of the activity by other enzymes may occur, such as in the case of glutathione-S-transferase GST enzymes [26,27].

The model proposed here, although preliminary and limited to a small number of gene polymorphisms investigated in this particular occupational field, might be useful to distinguish the relative risk among ethnic groups towards specific functional enzymes involved in the detoxification, protection from oxidative stress, and repair of damaged DNA pathways. Nonetheless, by PCA the model has been able to identify (1) similarity in the risk between ethnicities, (2) correlation of the ethnicity with specific metabolic genes, and (3) an estimation of the RR indicator in the four ethnic macro-populations. According to our results, it seems that South Asians have the cumulatively highest susceptibility risk compared to the other populations, while Africans have the lowest risk. 

Even though this model might be useful to obtain an indication of the susceptibility risk in different populations, it is still preliminary, may have some limitations, and could be improved. Among these limitations, it is worth mentioning (1) the restricted selection of a few enzyme categories with the exclusion of other enzymes; (2) the unknown role of novel or undiscovered variants, which might compensate for the missing function of enzymes with similar activity; and (3) the unpredictability of a combined effect when multiple variants coexist on the same gene. The model might also be improved, considering a larger number of individuals and gene polymorphisms to increase the statistical power of the analysis. However, these preliminary results are encouraging and might stimulate other investigators to explore this opportunity. To summarize, we believe the model might be further implemented by using a universal, reliable, and official SNP database that might be able to provide uniform data on the genotype frequency for all existing ethnicities. Once implemented, the proposed model might be exploited in the near future, as a useful tool to replace the laboratory analysis of susceptibility biomarkers in workers who may not wish to consent to blood sampling for large investigational and epidemiologic studies. This tool, taking into account the ethnic difference in the susceptibility of workers, might also contribute to confirming or updating the OELs in factories, where the ethical principle to protect the most susceptible individual is extended to the safety of all with no need to perform personalized genetic analyses.

## 5. Conclusions

In the last fifty years, the transcontinental migrations experienced in the European countries, including Italy, have influenced different aspects of the human health and well-being, from the re-appearance of extinct diseases; changes in diet, behavior, and the perception of health and safety; up to the change in environmental and occupational contexts. Individual lifestyle, habits, use of drugs, genetic background, and environmental exposures to toxicants represent a concern for humans, implying the need of a different and effective approach to the management of health risks.

Furthermore, recent climate change and massive immigration are contributing to the recrudescence of infective diseases like scabies and tuberculosis, as well as to the widespread diffusion of mosquito-hosted viruses that need to be faced in a short time as an imminent hazard for our health (i.e., Chikungunya, Dengue, and West Nile viruses) [28,29,30].

Despite the fact that genetic information is still being used in occupational risk assessment models, various ethical and social issues may arise when dealing with genetic analysis at the workplace. The reluctance to consent from immigrant and local workers, the expenses sustained by the research laboratory, and the necessity for approvals by the manufacturing company and local health units may not facilitate the success of the biomonitoring campaign, despite the importance of assessing the occupational exposure risk. Furthermore, the misunderstanding of “workers susceptibility”, if not correctly explained, might be interpreted as weakness or inability to accomplish a particular job task, which is in contrast to the main goal of genotyping, the role of which is to assess the impact of the genetic background of different populations on risk estimates. In fact, the main goal of gene–environment interaction studies, from early biological alterations to disease development and progression, taking into account other factors, such as one or more aspects of individual susceptibility, might integrate the conventional health risk assessment paradigm for chemical exposures; this allows the adoption of a comprehensive model, tailored to individual subjects or specific subpopulations. In the occupational setting, the most effective prevention strategy must take into account workers with the highest susceptibility, in order to guarantee safety and protection for all [31]. 

The predictive model proposed here may represent a potential but also perfectible alternative to the genotype screening of workers carried out at the molecular biology laboratory. Although the PCA reported here has been limited to three categories of biotransformation enzymes and four main ethnic populations, we believe it might be improved and extended to other ethnic sub-groups. By using this model, taking advantage of the genetic information publicly available on web databases, the genetic screening of workers might be avoided in the next future, at no cost for the research institute, with no need for trained staff and laboratory equipment, and no invasiveness or harm to the worker. 

When there is no access to genotyping tests or in the absence of informed consent from workers, the allele frequency of a known ethnicity is necessarily regarded as a useful indicator of the probable subject’s genotype of the ethnic group to which he or she belongs [15]. We have to be aware that continuous migrations with the admixture of populations, influence of diet, environmental exposure, and cultural behavior may modify the utility of ethnicity to predict an individual genotype [16]. Thinking in the very long term, we expect that polymorphic genes will continue to vary, and the variations recorded in public databases will help us to make use of the modified genotypes.

## Figures and Tables

**Table 1:** List of gene polymorphisms, grouped by functional activity and analyzed by principal component analysis (PCA).

Oxidative Stress	Single Nucleotide Polymorphism	rs Number (Rererence Single Nucleotide Polymorphism)
Nuclear factor (erythroid-derived 2)-like 2	NRF2	rs6721961 rs35652124
Heme-Oxygenase	HO-1	rs2071746
NAD(P)H Quinone Dehydrogenase 1	NQO1	rs1800566 rs1131341
Superoxide dismutase 2	SOD2	rs4880
**Detoxification**	**Single Nucleotide Polymorphism**	**rs Number**
Glutathione S transferase A1	GST-A1	rs3957357
Glutathione S transferase M1	GST-M1	rs366631
Glutathione S transferase T1	GST-T1	rs17856199
Glutathione S transferase P1	GST-P1	rs1695
Epoxide Hydrolase 1	EPHX1 Ex_3	rs1051740
Epoxide Hydrolase 1	EPHX1 Ex_4	rs2234922,
Cytochrome P450 1A1	CYP1A1_2A	rs4646903
	CYP1A1_2C	rs1048943
Cytochrome P450 2E1	CYP2E1*6	rs6413432
	CYP2E1*5B	rs3813867
Mieloperoxidase	MPO	rs2333227
**DNA repair**	**Single Nucleotide Polymorphism**	**rs Number**
X-Ray Repair Cross Complementing 1	XRCC1	rs25487
		rs13181
X-Ray Repair Cross Complementing 3	XRCC3	rs1799782
		rs861539
XP complementation group D	XPD/ERCC2	rs1799793
Excision repair protein	XPD/ERCC2	rs13181
8-Oxoguanine Glycosylase	hOGG1	rs1052133

**Table 2:** Relative risk of unfavorable SNP in the three groups of genes, detoxification, oxidative stress an DNA repair, for the four ethnicity groups. A cumulative risk, relative to the three groups of genes is also reported.

Ethnicity	Relative Risk			
	Detoxification	Oxidative	DNA Repair	Average
Africa	1	1	1	1
East Asia	1.9	10	3.2	5
Europe	2.5	1.3	19	7.6
South Asia	4.5	22.5	8	11.7

The relative risk of the four ethnic groups has been worked out for each cluster of gene polymorphisms.

## Supplementary Materials

The following are available online at https://www.mdpi.com/1660-4601/16/11/2012/s1, Table S1: Genotype and allele frequency of four main ethnic groups and relative risk estimation for the homozygous variant and heterozygous genotype.
