# Supplementary material for: A Predictive Model Assessing Genetic Susceptibility Risk at Workplace
Source: Int J Environ Res Public Health. 2019 Jun 5;16(11):2012. doi: 10.3390/ijerph16112012 (PMC6603935; doi:10.3390/ijerph16112012)
Supplement: Supplementary file 1 [file ijerph-16-02012-s001.pdf]

Supplementary Table 1. Genotype and allele frequency of four main ethnic groups and relative risk estimation for homozygousvariant and heterozygous genotype

Gene polymorphism	Population	Genotype Frequency			High Risk	Moderate Risk	Allele frequency	
		Homozygous wild type	Homozygous variant	Heterozygous	Homozygous variant Population/All	Heterozygous Populatiom/All	Major allele	Minor allele
NRF2 rs6721961 C617A (in the promoter)		**GG**	**TT**	**GT**	**TT**	**GT**	**G**	**T**
	African	0.887	0.002	0.112	0.069	0.483	0.943	0.057
	East Asian	0.577	0.063	0.359	2.172	1.547	0.757	0.243
**Oxidative Stress**	South Asian	0.726	0.031	0.243	1.069	1.047	0.848	0.152
	European	0.763	0.014	0.223	0.483	0.961	0.875	0.125
	All	0.739	0.029	0.232			0.855	0.145
NRF2 rs35652124 A653G (in the promoter)		**TT**	**CC**	**CT**	**CC**	**CT**	**T**	**C**
	African	0.720	0.015	0.265	0.091	0.626	0.852	0.148
	East Asian	0.208	0.312	0.480	1.902	1.135	0.448	0.552
**Oxidative Stress**	South Asian	0.207	0.286	0.507	1.744	1.199	0.460	0.540
	European	0.479	0.091	0.429	0.555	1.014	0.694	0.306
	All	0.413	0.164	0.423			0.624	0.376
NQO1 rs1800566 C609T or C559T (base transition in exon 6) (Pro^{187}Ser)		**GG**	**AA**	**AG**	**AA**	**AG**	**G**	**A**
	African	0.675	0.026	0.300	0.295	0.748	0.825	0.175
	East Asian	0.319	0.157	0.524	1.784	1.307	0.581	0.419
**Oxidative Stress**	South Asian	0.411	0.127	0.462	1.443	1.152	0.642	0.358
	European	0.626	0.048	0.326	0.545	0.813	0.789	0.211
	All	0.510	0.088	0.401			0.711	0.289
NQO1 rs1131341 C465T(Arg^{139}Trp)		**GG**	**AA**	**AG**	**AA**	**AG**	**G**	**A**
	African	0.994	0	0.006	0	0	0.997	0.003
	East Asian	0.960	0	0.040	0	1	0.980	0.020
**Oxidative Stress**	South Asian	0.910	0.010	0.080	5	2.051	0.950	0.050
	European	0.950	0	0.050	0	1.282	0.975	0.025
	All	0.959	0.002	0.039			0.979	0.021

HO-1 rs2071746 -413A>T		**AA**	**TT**	**AT**	**TT**	**AT**	**T**	**A**
	African	0.098	0.474	0.428	1.596	0.926	0.688	0.312
	East Asian	0.242	0.288	0.470	0.970	1.017	0.523	0.477
**Oxidative Stress**	South Asian	0.190	0.309	0.501	1.040	1.084	0.559	0.441
	European	0.306	0.187	0.507	0.630	1.097	0.440	0.560
	All	0.241	0.297	0.462			0.528	0.472
SOD2 rs4880G/A (exon 2) (Ala[16]Val)		**AA**	**GG**	**AG**	**GG**	**AG**	**A**	**G**
	African	0.333	0.180	0.487	0.923	1.127	0.576	0.424
	East Asian	0.764	0.014	0.222	0.072	0.514	0.875	0.125
	South Asian	0.245	0.262	0.493	1.344	1.141	0.492	0.508
**Oxidative Stress**	European	0.294	0.227	0.479	1.164	1.109	0.534	0.466
	All	0.373	0.195	0.432			0.589	0.411
GST-A1 rs3957357 C69T (non coding)		**GG**	**AA**	**AG**	**AA**	**AG**	**G**	**A**
	African	0.470	0.103	0.427	0.972	1.062	0.684	0.316
	East Asian	0.740	0.018	0.242	0.170	0.601	0.861	0.139
**Detoxification**	South Asian	0.440	0.131	0.429	1.236	1.067	0.654	0.346
	European	0.318	0.177	0.505	1.670	1.256	0.571	0.429
	All	0.492	0.106	0.402			0.693	0.307
GST-M1 rs366631							**Positive**	**Null**
	African						0.684	0.316
	East Asian						0.479	0.521
**Detoxification**	South Asian						0.597	0.403
	European						0.488	0.512
	All						0.520	0.480* (no Americans were included)
GST-T1 rs17856199							**Positive**	**Null**
	African						0.629	0.371
**Detoxification**	East Asian						0.524	0.476
	South						0.755	0.245

	Asian							
	European						0.867	0.183
	All						0,736	0.264* (no Americans were included)
GST-P1(319A>G) rs1695 Ile^{105}Val (missense mutation)		**AA**	**GG**	**AG**	**GG**	**AG**	**A**	**G**
	African	0.265	0.225	0.510	1.619	1.194	0.520	0.480
	East Asian	0.673	0.030	0.298	0.216	0.698	0.821	0.179
**Detoxification**	South Asian	0.513	0.102	0.384	0.734	0.899	0.706	0.294
	European	0.445	0.107	0.447	0.770	1.047	0.669	0.331
	All	0.434	0.139	0.427			0.647	0.353
EPHX1 Ex_3 rs1051740 Tyr^{113}His (slow allele)		**TT**	**CC**	**TC**	**CC**	**TC**	**T**	**C**
	African	0.744	0.026	0.230	0.222	0.585	0.859	0.141
	East Asian	0.272	0.236	0.492	2.017	1.252	0.518	0.482
**Detoxification**	South Asian	0.384	0.139	0.476	1.188	1.211	0.623	0.377
	European	0.487	0.095	0.417	0.812	1.061	0.696	0.304
	All	0.490	0.117	0.393			0.687	0.313
EPHX1 Ex_4 rs2234922 His^{139}Arg*(fast allele)*		**AA**	**GG**	**AG**	**AA**	**AG**	**A**	**G**
	African	0.421	0.127	0.452	0,673	1.426	0.647	0.353
	East Asian	0.780	0.016	0.204	1,246	0.644	0.882	0.118
**Detoxification**	South Asian	0.605	0.074	0.321	0,966	1.013	0.766	0.234
	European	0.686	0.014	0.300	1,096	0.946	0.836	0.164
	All	0.626	0.057	0.317			0.785	0.215
CYP1A1_2A rs4646903 (T> C mutation at 3′ UTR)		**AA**	**GG**	**AG**	**GG**	**AG**	**A**	**G**
	African	0.589	0.057	0.354	0.523	0.959	0.766	0.234
	East Asian	0.329	0.188	0.482	1.725	1.306	0.570	0.430
**Detoxification**	South Asian	0.446	0.125	0.429	1.147	1.163	0.661	0.339
	European	0.793	0.008	0.199	0.073	0.539	0.893	0.107
	All	0.522	0.109	0.369			0.707	0.293

CYP1A1_2C<br>rs1048943 Ile^{462}Val		**TT**	**CC**	**TC**	**CC**	**TC**	**T**	**C**
	African	0.986	0	0.014	0	0.07	0.993	0.007
	East Asian	0.562	0.065	0.373	1.625	1.995	0.748	0.252
**Detoxification**	South Asian	0.765	0.018	0.217	0.45	1.16	0.873	0.127
	European	0.930	0	0.070	0	0.374	0.965	0.035
	All	0.773	0.040	0.187			0.867	0.133
CYP2E1*6<br>***rs6413432 (7632 T>A)***(intron mutation)		**TT**	**AA**	**AT**	**TT**	**AT**	**T**	**A**
	African	0.855	0.009	0.136	1.199	0.538	0.923	0.077
	East Asian	0.536	0.079	0.385	0.752	1.522	0.728	0.272
**Detoxification**	South Asian	0.628	0.043	0.329	0.881	1.300	0.792	0.208
	European	0.805	0.016	0.179	1.129	0.708	0.895	0.105
	All	0.713	0.034	0.253			0.839	0.161
CYP2E1*5B<br>***rs3813867 (in the 5′ UTR)***		**GG**	**CC**	**CG**	**GG**	**CG**	**G**	**C**
	African	0.871	0.005	0.124	1.032	0.873	0.933	0.067
	East Asian	0.651	0.056	0.294	1	2	0.798	0.202
**Detoxification**	South Asian	0.982	0	0.018	1	0	0.991	0.009
	European	0.920	0.002	0.078	1.090	0.549	0.959	0.041
	All	0.844	0.014	0.142			0.915	0.085
MPO rs2333227 G463A (in the promoter region)		**CC**	**TT**	**CT**	**CC**	**CT**	**C**	**T**
	African	0.415	0.145	0.440	0.675	1.375	0.635	0.365
	East Asian	0.734	0.02	0.246	1.193	0.769	0.857	0.143
**Detoxification**	South Asian	0.763	0.016	0.221	1.241	0.691	0.873	0.127
	European	0.588	0.064	0.348	0.956	1.088	0.762	0.238
	All	0.615	0.065	0.320			0.775	0.225
XRCC3<br>rs1799782 Arg^{194}Trp		**GG**	**AA**	**AG**	**AA**	**AG**	**G**	**A**

	African	0.858	0.002	0.141	0.091	0.691	0.928	0.072
	East Asian	0.518	0.081	0.401	3.682	1.966	0.718	0.282
**DNA repair**	South Asian	0.783	0.004	0.213	0.182	1.044	0.890	0.110
	European	0.907	0.010	0.083	0.455	0.407	0.948	0.052
	All	0.774	0.022	0.204			0.876	0.124
XRCC3 rs861539 Thr²⁴¹Met (C/T transition at codon 241)		**GG**	**AA**	**AG**	**AA**	**AG**	**G**	**A**
	African	0.657	0.038	0.306	0.603	0.994	0.809	0.191
	East Asian	0.855	0.002	0.143	0.032	0.464	0.927	0.073
**DNA repair**	South Asian	0.630	0.049	0.321	0.778	1.042	0.790	0.210
	European	0.378	0.165	0.457	2.619	1.484	0.606	0.394
	All	0.629	0.063	0.308			0.783	0.217
XPD rs1799793 Asp^{312}Asn (XPD G312A)		**CC**	**TT**	**CT**	**TT**	**CT**	**C**	**T**
	African	0.858	0.005	0.138	0.098	0.481	0.927	0.073
	East Asian	0.903	0.002	0.095	0.039	0.331	0.950	0.050
**DNA repair**	South Asian	0.440	0.110	0.450	2.157	1.568	0.665	0.335
	European	0.406	0.123	0.471	2.412	1.641	0.641	0.359
	All	0.662	0.051	0.287			0.806	0.194
XRCC1 rs 25487 Arg^{399}Gln		**CC**	**TT**	**CT**	**TT**	**CT**	**C**	**T**
	African	0.787	0.008	0.206	0.103	0.564	0.890	0.110
	East Asian	0.587	0.058	0.355	0.744	0.973	0.765	0.235
**DNA repair**	South Asian	0.436	0.123	0.442	1.577	1.211	0.656	0.344
	European	0.402	0.133	0.465	1.705	1.274	0.634	0.366
	All	0.557	0.078	0.365			0.740	0.260
hOGG1 rs1052133 Ser^{326}Cys		**CC**	**GG**	**CG**	**GG**	**CG**	**C**	**G**
	African	0.726	0.036	0.238	0.313	0.638	0.845	0.155
	East Asian	0.220	0.315	0.464	2.739	1.243	0.452	0.548
**DNA repair**	South Asian	0.415	0.127	0.458	1.104	1.228	0.644	0.356

	European	0.624	0.030	0.346	0.261	0.928	0.797	0.203
	All	0.511	0.115	0.373			0.698	0.302
XPD rs13181 Lys^{751}Gln (T > G)		**TT**	**GG**	**GT**	**GG**	**GT**	**T**	**G**
	African	0.651	0.035	0.315	0.530	0.924	0.808	0.192
	East Asian	0.851	0.004	0.145	0.061	0.425	0.924	0,076
**DNA repair**	South Asian	0.421	0.115	0.464	1,742	1.361	0.653	0,347
	European	0.417	0.145	0.437	2.197	1.282	0.636	0.364
	All	0.593	0.066	0.341			0.763	0.237
XRCC1 rs25487 Arg^{194}Trp		**TT**	**CC**	**CT**	**CC**	**CT**	**C**	**T**
	African	0.008	0.787	0.206	1.413	0.564	0.890	0.110
	East Asian	0.058	0.587	0.355	1.054	0.973	0.765	0.235
	South Asian	0.123	0.436	0.442	0.783	1.211	0.656	0.344
	European	0.133	0.402	0.465	0.722	1.274	0.634	0.366
	All	0.078	0.557	0.365			0.740	0.260

All the genes conferring a specific (unfavourable) susceptibility are those listed in the homozygous variant genotype column.

Four genes highlighted in bold *Italics* i.e. EPHX1 rs2234922, MPO rs2333227, CYP2E1*6 rs6413432, CYP2E1*5B rs3813867indicate polymorphisms whose homozygous wild-type genotype might confer a disadvantageous condition in the enzymatic function.
